# Sequence effects during speech perception reveal multi-accent processing costs

**DOI:** 10.3758/s13414-025-03220-5

**Published:** 2026-02-23

**Authors:** Drew J. McLaughlin, Jackson S. Colvett, Julie M. Bugg, Kristin J. Van Engen

**Affiliations:** 1 Basque Center on Cognition, Brain and Language, Paseo Mikeletegi, 69, 20009 Donostia-San Sebastián, Gipuzkoa Spain; 2 Department of Psychological & Brain Sciences, Washington University in St. Louis, St. Louis, MO USA; 3 Department of Psychological Science, Berry College, Mt. Berry, GA USA

**Keywords:** Pupillometry, Speech processing, Congruency sequence effect, Accent

## Abstract

Alternating between different talkers during listening typically incurs a cognitive processing cost. How these processing costs manifest, and potentially differ, in a multi-accent setting remains to be examined. Across two experiments, we investigate (1) whether talker and accent switching costs are driven by engagement of a recalibration mechanism, and (2) whether global listening context affects the magnitude of talker and accent switching costs. The results of our first experiment indicate that switching between speakers of the same second language (L2) accent (e.g., between two Mandarin-accented speakers of English) was less cognitively challenging than switching between speakers of different L2 accents (e.g., between a Mandarin-accented speaker and a Turkish-accented speaker of English). This outcome suggests that the perceptual distance (i.e., the holistic estimate of spectral and temporal differences in acoustic signals) between two speakers’ productions determines the size of associated switching costs, such that recalibration is less cognitively demanding for speakers with the same L2 accent. In our second experiment, we examine whether a more challenging block-wide listening context results in a global upregulation of cognitive resources, and, subsequently, reduces the cognitive resources required to (a) process L2 accent and (b) resolve local talker and accent changes. Here, the overall cognitive demands of processing L2 accent were reduced, as predicted, but talker and accent switching costs were not. We conclude that talker and accent switching are supported by a recalibration mechanism and that global upregulation of cognitive resources may reduce L2 accent processing costs but not local switching costs.

**Supplementary Information:**

The online version contains supplementary material available at 10.3758/s13414-025-03220-5.

Imagine yourself sitting down for dinner with a group of friends. As the conversation switches from person to person, the cognitive systems that underlie speech processing need to accommodate the variability in each individual talker’s productions. Although conversing typically feels effortless to interlocutors, prior work indicates that alternating between different talkers during listening incurs a cognitive cost (referred to in prior work as a multi-talker processing cost; Martin et al., 1989; Mullennix et al., 1989; Choi et al., 2018; Choi & Perrachione, 2019; Heald & Nusbaum, 2014), suggesting that accommodation of idiosyncratic differences in talker productions may require engagement of specific cognitive mechanism(s). For example, listeners are typically slower to respond and less accurate in experimental blocks where multiple speakers are intermixed trial-to-trial compared with blocks with a single speaker (Kapadia & Perrachione, 2020). Now consider that one of your friends speaks with an L2 (i.e., second language) accent, and how this factor may exacerbate the challenges of a listening setting with multiple talkers. Even when an L2 speaker is highly proficient and intelligible, it has been shown that there can be an additional cognitive cost for processing L2-accented speech compared with L1- (first language) accented speech (Brown et al., 2020; McLaughlin & Van Engen, 2020). Would understanding that friend’s speech be easier if a different friend with the same accent spoke directly before them? What if the majority of speakers at that table spoke with an L2 accent?

The initial evidence surrounding these questions indicates that the processing costs associated with switching between talkers are even greater when switching from an L1 accent to an L2 accent (McLaughlin et al., 2023). It is possible that switching costs scale with the perceptual distance between talkers’ productions, such that each speaker’s accent type contributes to the magnitude of the switching cost. In the present study we define perceptual distance between speakers and accents as the holistic estimate of spectral and temporal differences in acoustic signals; this estimate captures spoken language variance at both the segmental and suprasegmental levels of linguistic units. We operationalize perceptual distance using a machine learning analysis of acoustics (see Supplemental Materials B); speakers of the same L2 accent (e.g., a Turkish accent and a Turkish accent) are closer than those of different L2 accents (e.g., a Turkish accent and a Mandarin accent). One theoretical framework that may account for prior data is an *active control model.* On this view, the processing costs associated with switching between talkers may be attributable to the “active control” (working memory and attentional control) required to accommodate acoustic differences across different talkers’ productions (Heald et al., 2016; Magnuson & Nusbaum, 2007; Nusbaum & Magnuson, 1997). More specifically, this account proposes a recalibration mechanism that supports rapid talker accommodation (i.e., following a switch in talkers) by mapping the current talker’s idiosyncratic productions to a listener’s phonological space.

The active control model also integrates easily with an exemplar model of speech perception, which proposes that episodic traces are encoded in the lexicon (Goldinger, 1998; Hintzman, 1984; Johnson, 1997, 2006; Pierrehumbert, 2001). On this view, listeners would create abstracted categories over time that link various cues (including nonlinguistic, social cues) to linguistic patterns. Ultimately, the knowledge amassed from exemplars would support top-down speech processing; for example, upon seeing a familiar face, such as that of a spouse, the listener ought to be able to retrieve their prior knowledge (a “talker-percept mapping,” as proposed by Magnuson, 2018; cf. Magnuson et al., 2021) and then implement top-down calibration of their perceptual categories to better match the speaker’s idiosyncratic speech. Figure 1 shows a diagram of how talker/accent recalibration may be integrated with detection of talker/accent changes during speech processing (diagram adapted from Magnuson, 2018).

**Fig. 1:**
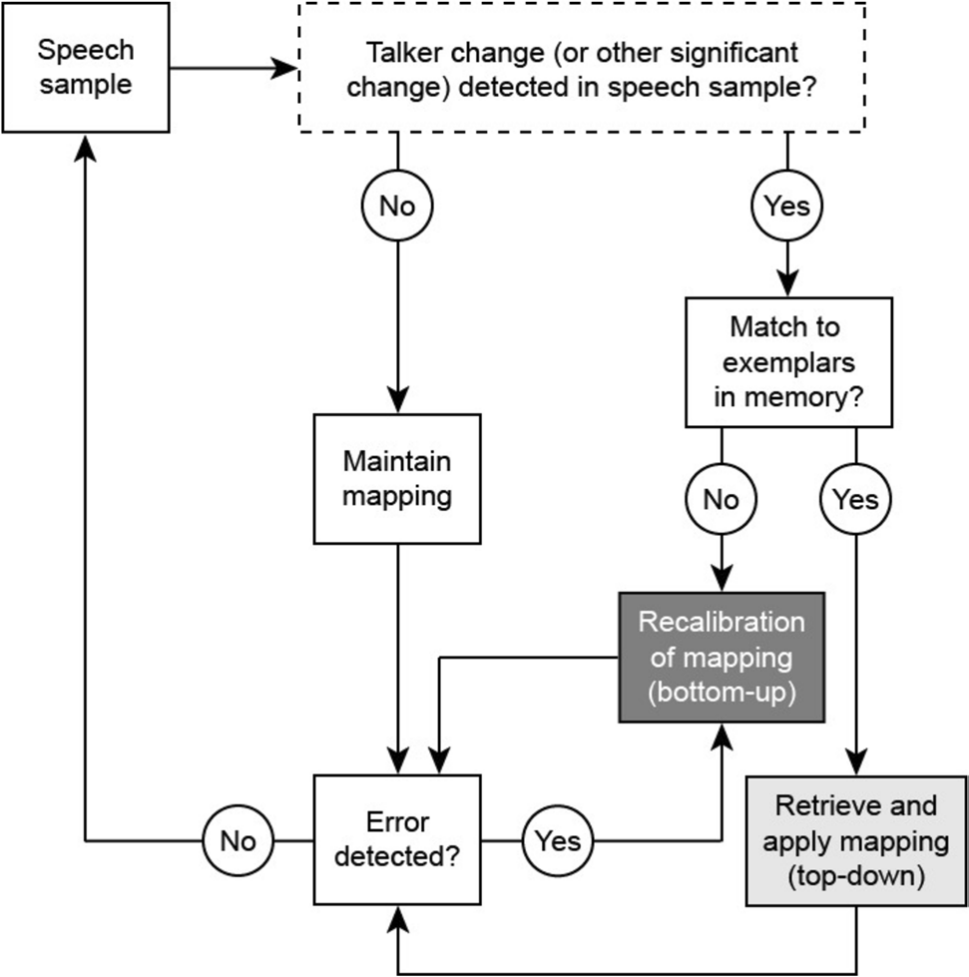
Diagram integrating hypotheses surrounding talker (and/or accent) switching costs and talker (and/or accent) recalibration, adapted from Magnuson (2018). Gray boxes show points of increased demands on working memory and attentional control (i.e., above baseline cognitive load expected for speech processing). The dark-gray box denotes the recalibration process, which adapts distributional categories based on bottom-up information from the speech sample (while the sample is held in working memory). The light-gray box denotes application of top-down knowledge pulled from memory. The top box (shown with dashed lines) is hypothesized to be a separate source of additional listening costs (see discussion of the *auditory streaming framework*). The terminology “mapping” follows Magnuson (2018) but may be more broadly interpreted as distributional knowledge of cues amassed from exemplars. For example, identification of a /p/ versus /b/ phoneme is informed by many distributions, including voice onset time, talker F0, and length of the following vowel

Several neuroimaging and behavioral findings support an active control model. Using functional magnetic resonance imaging (fMRI), Wong and colleagues (2004) found greater activation in the medial and superior temporal regions (areas associated with working memory; Paulesu et al., 1993) and in the superior parietal region (an area associated with attentional control; Fernandez-Duque & Posner, 2001; Shomstein, 2012) for mixed-talker blocks as compared with single-talker blocks. Their data align with an interpretation of talker-switching costs in which the incoming speech signal is held in working memory while a phonological mapping is calculated. Further evidence that working memory plays a key role in managing trial-to-trial talker changes comes from an examination of individual differences in working memory capacity and attentional control (McLaughlin et al., 2026). In that study, only working memory capacity predicted switching costs, such that listeners with greater capacities had reduced switching costs as compared with listeners with smaller capacities.

While the active control model accounts for many previous findings, additional mechanisms could provide alternative or complementary explanations of multi-talker processing costs. An additional (not mutually-exclusive) account of multi-talker processing costs is the *auditory streaming framework* (Shinn-Cunningham, 2008), which proposes that the processing costs associated with speaker changes stem from disruption of auditory attention. Specifically, processing costs are incurred when a listener is forced to shift their attention from one auditory object (a speaker) to another (Mehraei et al., 2018), as opposed to being incurred by calculating a phonological mapping. In the context of the diagram shown in Fig. 1, the costs associated with disruption of auditory attention would occur when the talker change is detected (i.e., when a “Yes” outcome from the dashed box occurs). Key evidence favoring this account comes from Kapadia and Perrachione (2020) who compared talker-switching costs during blocks in which the upcoming changes were and were not predictable. Results indicated an equivalent cost for any block with multiple talkers, even when changes in talkers were completely predictable (i.e., consistently changing back and forth between two talkers). This outcome aligns with an auditory streaming framework of talker-switching, in which any auditory change would be disruptive and costly (even for an experienced listener), though it is not necessarily at odds with the active control model. As shown in Fig. 1, the costs triggered by monitoring talker changes may occur in a separate stage from the costs associated with calibration to a talker and/or accent.

Processing costs can be reduced by supporting re-orientation of attention with a cue (such as the article “a” preceding a noun), although, in line with predictions of the auditory streaming framework, cues do not remove costs entirely. Choi et al. (2022) manipulated the presentation of an article (“a”) prior to each stimulus in a multi-talker experiment. When words were preceded 300 ms by the article “a” (spoken by the talker of the upcoming stimulus), processing costs were reduced. Additionally, processing costs were reduced further when the article was presented 600 ms prior to the word. However, for intervals greater than 600 ms (examined parametrically up to 1,500 ms), no additional benefits were observed. In other words, even an extremely early cue to an upcoming talker change did not entirely remove the processing costs of switching between talkers. It may be the case that early cues can mitigate the disruption of auditory attention but not the costs stemming from recalibrating perceptual categories to the talker.

The processing costs associated with switching between multiple talkers have also been examined with electroencephalography (EEG) and pupillometry (Lim et al., 2021). Using concurrent EEG and pupillometry (an index of cognitive load; Beatty, 1982), Lim and colleagues examined the costs associated with performing a delayed recall digit span task for single- versus mixed-talker blocks. The pupillometry data indicated a larger task-evoked response for the mixed-talker blocks compared with the single-talker blocks, indexing greater cognitive load in blocks with alternating talkers. The EEG data indicated an increased P3a-like neural response in multi-talker blocks, a component that has been linked to attentional reorientation (Polich, 2007). Additionally, the authors examined power in the alpha band for each block during encoding (i.e., while digits were presented to the listener) and retention (i.e., a delay period before recall). Here, one would predict greater activity for more challenging listening conditions that rely on working memory (Wilsch & Obleser, 2016). Opposite of predictions based on the active control model, there was no difference in alpha band power during encoding, and less power for multi-talker blocks during retention than single-talker blocks. That is, the increased P3a-like neural response observed for multi-talker blocks was attributed to disruptions in auditory streaming (i.e., forcing the listener to change attention from one speaker to another), and the lack of difference in alpha power was attributed to similar demands on working memory across multi-talker and single-talker blocks.

Evidence that talker switching costs may be exacerbated by differences in talker accent was found in McLaughlin et al. (2023). Using pupillometry as a physiological index of cognitive processing load, the authors examined speech processing in a multi-talker block with talkers of L1 (American English) and L2 (Mandarin) accents. The authors assessed the costs of switching between talkers and between accents by examining the current trial (*N*) in context of the previous trial (*N* − 1). Pupil responses were larger when the speaker on trial *N* − 1 differed from the speaker on trial *N*, indicating greater cognitive processing load. Moreover, switching from an L1-accented speaker to an L2-accented speaker (“across-accent”) was more challenging than switching between two L2-accented speakers of the same accent (“within-accent”). One interpretation of the outcomes of McLaughlin et al. (2023) is that the perceptual distance between the productions of the previous and the current talker may determine the costs of accommodation. Following the diagram shown in Fig. 1, the greater cost for “across-accent” switches than “within-accent” switches might be explained by the differential demands placed on the recalibration mechanism. However, an alternative interpretation of these differential costs could not be ruled out due to the design of the task: Rather than costs scaling with the perceptual distance between the former and current talker, it may be the case that merely transitioning from an easier (L1 accent) to a more challenging (L2 accent) listening condition instigates a greater allocation of cognitive resources to speech processing.

## The current study

The aims of the current study, addressed in two experiments, respectively, were as follows: (1) Determine whether the source of across-accent switching costs is driven by engagement of a recalibration mechanism or accent difficulty and (2) investigate the potential impact of global listening context on within- and across-accent switching costs. Because the motivation for the latter aim (of Experiment 2) stems from the results of Experiment 1, we provide a brief overview of these aims here and reserve the detailed overviews (i.e., of research questions and hypotheses) for the presentations of each respective experiment.

To address the two competing explanations of the across-accent switching costs observed in McLaughlin et al. (2023), we first investigate multi-talker processing costs in a setting with multiple L2 accents. Specifically, in Experiment 1 we examine switching costs for two L2 accents that are matched for overall difficulty (as determined by intelligibility and a perceptual distance analysis, see Supplemental Materials). Although the L2 talkers in McLaughlin et al. were highly intelligible, the L2 accent condition was much more challenging and, thus, elicited a larger overall pupil response than the L1 accent condition (see also Brown et al., 2020; McLaughlin & Van Engen, 2020). By presenting two L2 accents of similar difficulty in Experiment 1, we will be able to isolate the potential role of an active control (i.e., recalibration) mechanism.

The design of our second experiment is largely influenced by work that has examined global adjustments of resource allocation induced by block-wide contexts (i.e., proportion effects; Bugg, 2014; Bugg & Gonthier, 2020; Logan & Zbrodoff, 1979; Siqi-Liu & Egner, 2020). In short, those designs manipulate the overall likelihood of certain types of trials (e.g., incongruent trials vs congruent trials in a Stroop paradigm; task switches vs. task repetitions in a task switching paradigm) across a block of trials, then assess how participants allocate cognitive resources in response to block-wide demand. To our knowledge, the speech perception literature has yet to assess whether listeners make similar adjustments, such that a block-wide listening context may affect cognitive resource allocation. Specifically, would the cognitive demands of L2 accent perception differ in a block-wide context where a second L2 accent is likely (i.e., as in Experiment 1 of the present study) versus a context where an L1 accent is likely (i.e., as in McLaughlin et al., 2023)? Similarly, it remains to be seen whether global upregulation may subsequently reduce the cognitive resources required to resolve local talker and accent changes.

## Experiment 1

In Experiment 1, we investigated whether switching between highly intelligible speakers with two different L2 accents (e.g., from a Mandarin-accented speaker of English to a Turkish-accented speaker of English) was more cognitively demanding than switching between speakers with the same L2 accent (e.g., from one Mandarin-accented speaker to a different Mandarin-accented speaker). We proposed two competing accounts: The *accent difficulty account* and the *recalibration distance account*. Where relevant, we note how the mechanisms proposed in the active control model and auditory streaming framework are interconnected with these two accounts.

The *accent difficulty account* predicts that, for two L2 accents of equal difficulty, there will be no difference in the cognitive demands associated with within-accent switches and across-accent switches. The outcomes of McLaughlin et al. (2023), which contained an L1 accent of lesser difficulty and an L2 accent of greater difficulty, found that across-accent switches from the L1 to L2 accent were more cognitively demanding than within-accent switches, while across-accents switches from the L2 to L1 accent were not. The accent difficulty account proposes that transitioning from an easier (L1 accent) to a more challenging (L2 accent) listening condition induces a greater demand on cognitive resources, whereas the opposite induces no additional cost. In a within-accent switch trial (from one L2-accented speaker to another) the listener is already prepared to process L2 accent, while in an across-accent switch trial (from an L1-accented speaker to an L2-accented speaker) the listener has to actively engage these resources—thus resulting in greater overall cognitive demands on these trials. Critically, in McLaughlin et al. (2023), the L2 accent was dramatically more difficult than the L1 accent. In the present study, the two L2 accents will be of similar difficulties, such that the accent difficulty account predicts that across-accent switches will not induce additional cognitive demands over within-accent switches.

The *recalibration distance account* predicts that across-accent switches will be more cognitively demanding than within-accent switches, even when the two accents are of equal difficulty. Under this account, the perceptual distance between two talkers’ productions determines the size of associated switching costs, because recalibration is more complex (i.e., as predicted by an active control model). This account predicts that switching between speakers will always incur a cost, and that the type of accent on trial* N* and trial *N* − 1 affects the degree to which switching is costly. Switching between speakers of the same accent (e.g., two Mandarin-accented speakers) will be easier than switching between speakers of different accents (e.g., Mandarin- and Turkish-accented speakers) because of the increased perceptual distance. Even if two accents are equally difficult at baseline, shifting to attend to the features of a new accent should be more difficult than hearing the same accent (i.e., where the acoustic space is more similar to that of the last trial, see Supplemental Materials B). Thus, it is possible that switching between speakers of two different L2 accents, as in Experiment 1, will incur greater cognitive costs than switching between speakers of the same L2 accent. Notably, the interaction observed in McLaughlin et al. (2023) does not necessarily support this account; across-accent switches from the L2 to L1 accent were not more cognitively demanding than within-accent switches, despite the perceptual distance being identical to an L1 to L2 across-accent switch. However, one explanation for this exceptional case might be that L1 accent is afforded a “familiarity benefit.” In the context of Fig. 1, a well-established mapping (based on a high density of exemplars) may be retrieved for the L1 accent, thereby reducing need for recalibration.

### Method

Materials, data, and analysis code for both experiments are available online (https://osf.io/gd8rp/). Preregistration of Experiment 1 is available online (https://osf.io/8y4hg). The recruitment plan and protocol for this experiment was approved by the Washington University in St. Louis Institutional Review Board.

#### Participants

Fifty-seven young adult participants (42 women, 14 men, one preferred not to answer; Age *M* = 19.25, *SD* = 1.23) were recruited from Washington University’s psychology participant pool and participated for course credit. After removing participants who were excluded due to experiment or equipment malfunction (three participants), blinking-related data loss (one participant), and excessive sleepiness (two participants), 51 participants were retained in the final sample (39 women, 11 men, one preferred not to answer; Age *M* = 19.20, *SD* = 1.22). The preregistered target sample size for both experiments (i.e., 50 participants) was selected based on sufficient power to detect effects in McLaughlin et al. (2023). All subjects were L1 speakers of English (of an American dialect) with normal hearing, normal (or corrected-to-normal) vision, and reported minimal exposure to Mandarin Chinese and Turkish (e.g., did not live with a speaker of either of those languages or study either of those languages in school). All participants reported learning at least one additional language in school; of the 51 total participants, approximately 65% reported learning their additional language before or at the age of 12 and approximately 20% reported learning their additional language before or at the age of 5 (at home).

#### Materials

Stimuli for Experiment 1 included recordings of two Mandarin Chinese-accented speakers of English and two Turkish-accented speakers of English drawn from SpeechBox (Bradlow, n.d.). Speakers were initially selected based on the researchers’ impression of accent strength and intelligibility, then confirmed to be appropriate for the research design post hoc with a perceptual distance analysis (Supplemental Materials) and intelligibility measures. Target sentences (*n* = 100) for the stimuli come from the Hearing in Noise Test (HINT; Nilsson et al., 1994), and were all semantically and syntactically normal.^1^ All speakers were the same sex to prevent undesired additional perceptual distance between talkers. The specific L2 accents selected (Mandarin and Turkish) and the sex of the speakers (men) was based on stimuli availability. The Mandarin, Cantonese, and Turkish L2-accented English materials in the SpeechBox archive are the most abundant (with 14, 14, and 11 speakers, respectively); using Mandarin and Turkish speakers rather than Mandarin and Cantonese speakers was based on the assumption that this would emphasize across-accent perceptual distance. The majority (*n* = 22) of the Mandarin- and Turkish-accented speakers in the archive are men (versus *n* = 5 women speakers), thus providing the most options for selecting talkers of similar qualities and difficulty.

#### Procedure

The procedure for Experiment 1 was adapted from previous work assessing pupil response for intelligible accented speech (e.g., McLaughlin & Van Engen, 2020; McLaughlin et al., 2023). Participants entered the testing room and the instructions were delivered via a video call. Participants wore circumaural headphones and rested their chins on a head-mount that was 90 cm away from a 53.5 cm × 30 cm computer screen. All equipment was positioned following EyeLink specifications. A 9-point calibration and validation procedure was conducted for all subjects before they began the task.

Participants were instructed to fixate on a centrally presented cross throughout the experiment. The cross changed color to indicate whether participants were in a section of the trial where they should reduce their blinking or one where they could blink freely. When the cross was red, participants were instructed to reduce blinking as much as they were comfortable and to attend to the auditory stimulus. When the cross was blue, participants were instructed to blink as much as they would like. Each trial began with a baseline period of 3000 ms of silence and a red cross. Next, with the red cross still present, the stimulus played followed by a delay period of 3,000 ms. At this point, the color of the cross changed to blue, indicating that subjects could blink freely. Participants were instructed to repeat the sentence they heard out loud. Participants’ responses were recorded as part of the ongoing video call. Finally, participants pressed the spacebar to move to the next trial, and a 3000 ms silent delay period with a blue fixation cross was presented. The delay allowed the pupil response to recover between trials.

The experimental blocking followed Experiment 2 of McLaughlin and colleagues (2023).

Participants first completed a four-trial practice phase in which each of the four speakers used in the main experiment was presented once. The practice trials followed the same trial procedure as the experimental task. Following the practice block, participants completed four 25-trial blocks. Each block began with a start trial, which was excluded from analysis as it was neither a repeat trial nor a switch trial. Each of the four speakers was the start trial in one of the four blocks. The remaining 24 trials of each block comprised six trials from each of the four speakers. The trial order was pseudorandom, such that the participant could not predict what speaker would be used on the next trial. Each block contained eight no switch trials (i.e., speaker from trial *N* − 1 spoke on trial *N*), eight within-accent switch trials (i.e., speaker from trial *N* − 1 was a different speaker than the speaker on trial *N*, but both speakers had the same accent) and eight across-accent switch trials (i.e., the speaker on trial *N* − 1 was a different speaker and had a different accent than the speaker on trial N). For each of these three conditions, we also assessed whether the effects differed by the accent on trial N. Between each block, participants took a self-timed break in which participants were instructed not to leave the chair or remove their head from the headrest.

After all four blocks were complete, participants completed language and demographic questionnaires and were debriefed on the task. The experiment took approximately 45 minutes to complete.

##### **Pupillometry data preparation**

Data were pre-processed in R (version 4.0.4 “Lost Library Book”) using a combination of tools in the *gazeR* package (Geller et al., 2020) and the *dplyr* package (Wickham et al., 2023). Standard pupillometry procedures were followed. First, blinks (areas of missing data) were identified, and participants with more than 20% data loss due to blinking were excluded from the sample. These blink windows were next expanded 100 ms prior and 200 ms following (to remove extraneous values that are logged when the eyelid is partially obscuring the pupil), and missing data was replaced using linear interpolation. A 5-point moving average was next applied for data smoothing.

For the baselining (alignment) of each trial, the median pupil diameter during the 500 ms immediately preceding stimulus onset of each trial was calculated. Subtractive baselining was used (Reilly et al., 2019), meaning that the baseline value was subtracted from all other values within the trial. The final processing step involved time-binning the data to reduce the sampling frequency from 500 Hz to 50 Hz.

##### **Intelligibility data preparation**

Repetitions of the target sentences were scored as proportions of the keywords correctly identified, where keywords included all sentence items except “the” and “a.” Differences in plurality and verb tense (specifically differences in use of -ed morpheme) were allowed. The Mandarin-accented speakers were 94% and 93% intelligible and the Turkish-accented speakers were 98% and 99% intelligible.

##### **Analysis window selection**

To avoid increasing researcher degrees of freedom during the analysis process (see Peelle & Van Engen, 2021), we selected our analysis window before viewing effects of accent and switch. The only data viewed prior to window selection was a single plotted curve summarizing the mean of all trial and subject data (i.e., to confirm a polynomial analysis was appropriate). Due to the delay of the pupil response, which is typically 200–300 ms, we opted to begin our analysis window at 300 ms after target onset. The end of the analysis window was set to 1,000 ms after the average offset time of the stimuli (2,680 ms).

##### Growth curve analysis

Pupil data was modeled with the *lme4* R package (Bates et al., 2014) following a growth curve analysis approach (similar to polynomial regression; Mirman, 2016). Orthogonalized polynomial predictors (linear, quadratic, cubic) were incorporated into the fixed and random effects of the model, allowing for a non-linear time-course analysis suitable for the pupillometry data. In growth curve analysis, the estimates of condition fixed effects reflect vertical differences in overall magnitude between levels, and interactions between these condition fixed effects and the polynomial parameters determine whether the shape of the pupil response differs by condition. We used a random effect structure that included random slopes of the linear, quadratic, and cubic polynomials from the growth curve analysis model along with the standard random intercepts for subjects and items.

### Results

All log-likelihood model comparisons from the growth curve analysis of Experiment 1 are reported in Table 1. The shape of the task-evoked pupil response was fit using linear, quadratic, and cubic polynomials (all significantly improved fit; *p*s < .001). The effects of intelligibility (proportion of words correctly perceived) and trial (a proxy for fatigue/habituation of the pupil response; see McLaughlin et al., 2023) both accounted for a large amount of variance (*p*s < .001), and were thus included in the model. Trials with poorer intelligibility scores had larger amplitudes (*ß* = −39.41, *p* < .001) and there was a general decrease in pupil response amplitude across the task (*ß* = −1.56, *p* < .001).

**Table 1:** Log-likelihood model comparisons for growth curve analysis of Experiment 1

Effect	*χ*^2^	*df*	*p*
Linear polynomial	8186.00	1	<.001
Quadratic polynomial	4288.50	1	<.001
Cubic polynomial	227.46	1	<.001
Intelligibility	163.48	1	<.001
Trial	13421.00	1	<.001
Accent	651.66	1	<.001
Switch	52.01	2	<.001
Accent : Switch	51.99	2	<.001
Accent : Linear polynomial	600.18	1	<.001
Accent : Quadratic polynomial	19.82	1	<.001
Accent : Cubic polynomial	10.64	1	.001
Switch : Linear polynomial	40.33	2	<.001
Switch : Quadratic polynomial	10.66	2	.005
Switch : Cubic polynomial	1.40	2	.50
Accent : Switch : Linear polynomial	77.44	2	<.001
Accent : Switch : Quadratic polynomial	15.30	2	<.001
Accent : Switch : Cubic polynomial	8.64	2	.01

Colons indicate interactions. Levels of accent included Mandarin (reference) and Turkish. Levels of switch included no switch (reference), within-accent switch, across-accent switch.

**Fig. 2:**
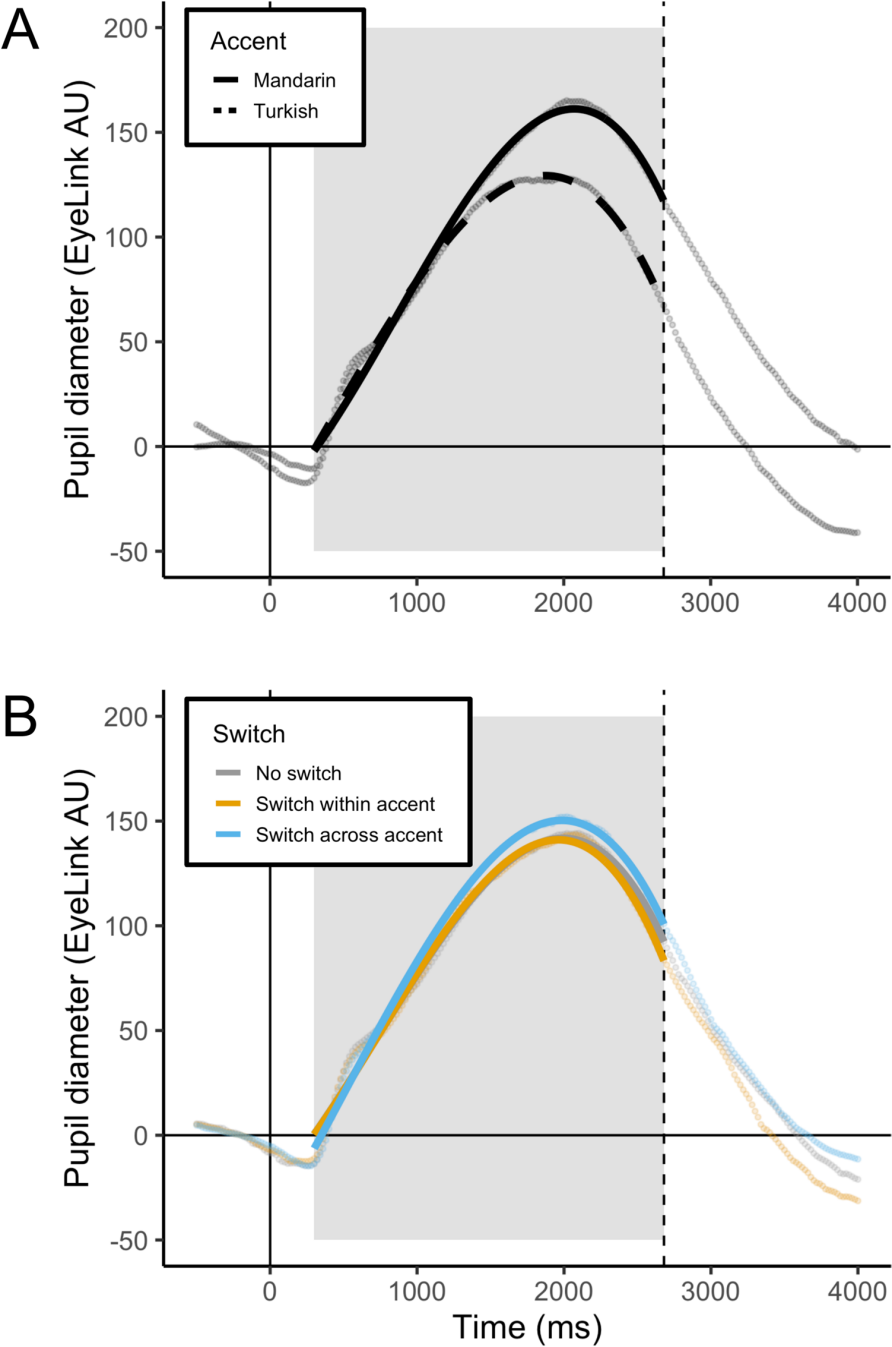
The effects of accent (**A**) and switch (**B**) are shown with raw data points and predicted fit lines. The solid vertical line at zero indicates sentence onset and dashed vertical line indicates average sentence offset. The gray box denotes the area of data used for analyses. (Color figure online)

The fixed effects of interest were accent (levels: Mandarin, Turkish) and switch (levels: no switch, within-accent switch, across-accent switch), as well as the interactions between these effects and the polynomial time (i.e., shape) terms. Before adding interactions with the model shape parameters, the estimate of the effect of accent indicated a smaller overall amplitude for the Turkish accent condition than the Mandarin accent condition (*ß* = −19.72, *p* < .001). As shown in Fig. 2A, the peak of the Mandarin accent condition was greater than the peak of the Turkish accent condition. For the effect of switch, before adding the interactions with the model shape parameters, the model estimate indicated a larger overall amplitude for both the within-accent switch (*ß* = 1.91, *p* = .04) and across-accent switch (*ß* = 6.54, *p* < .001) conditions, as compared with the no switch condition; rotation of the dummy-coded levels of switch revealed that the across-accent switch condition also had a larger overall amplitude as compared with the within-accent switch condition (*ß* = 4.64, *p* < .001). However, the effect of switch interacted with two of the polynomial time terms (linear: *p* < .001; and quadratic: *p* = .005), indicating differences in the shape of the pupil response between switch conditions. Notably, when conducting GCA we typically see a faster increase in pupil response (reflected in the linear term) for curves with an overall higher peak pupil response. Model estimates for the within-accent switch condition indicated that, as compared with the no switch condition, there was not a significant difference in the rate of increase of the pupil response, but there was a significantly sharper peak (*ß* = −23.08, *p* = .02). Together, these outcomes indicate that while there may be differences in the intercept and the timing of the return to baseline between the no switch and within-accent switch conditions, the peak pupil response amplitudes were statistically the same, as seen in Fig. 2B. The across-accent switch condition, on the other hand, showed a faster increase in the pupil response (*ß* = 44.88, *p* < .001) in combination with a sharper peak (*ß* = −32.43, *p* = .001), reflecting an overall greater peak pupil response than the no switch condition.

**Fig. 3:**
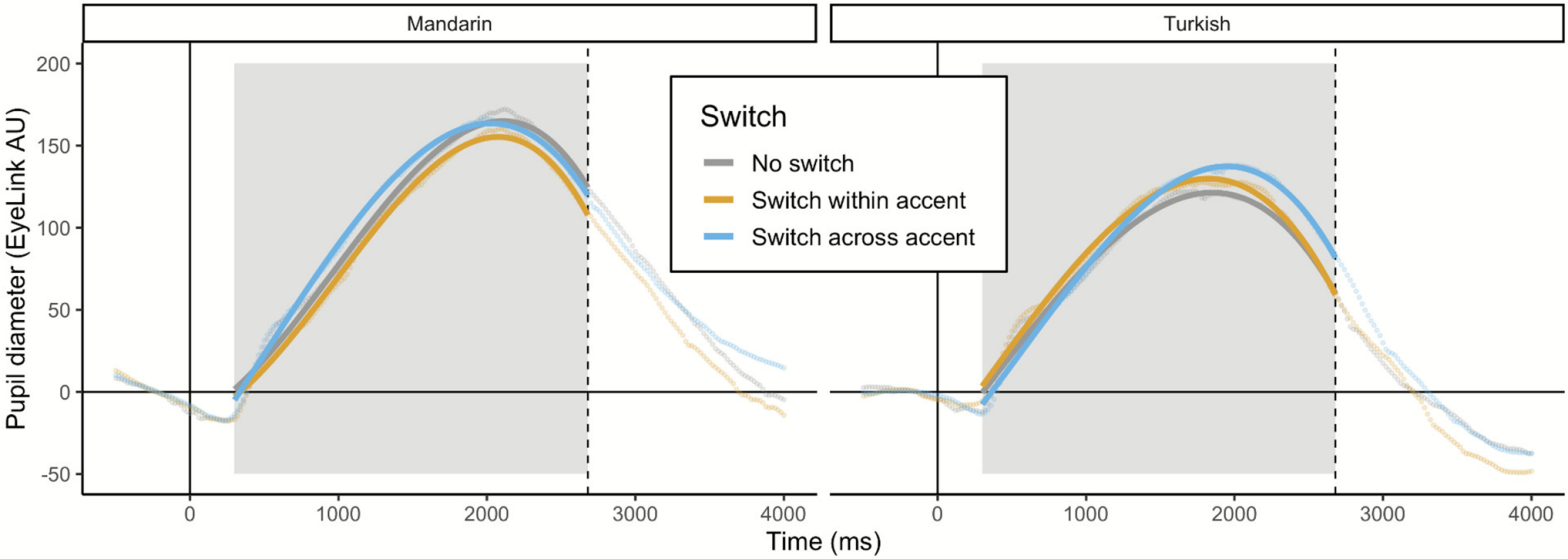
The interaction of accent and switch is shown with raw data points and predicted fit lines. The solid vertical line at zero indicates sentence onset and dashed vertical line indicates average sentence offset. The gray box denotes the area of data used for analyses. (Color figure online)

We further tested the interaction between accent and switch, as well as the three-way interaction of accent and switch with each of the polynomial terms. All interactions significantly improved model fit (all *p*s < .05). Figure 3 displays the interaction between accent and switch with predicted fits from the full model. To best understand the nature of the accent by switch interaction we examined the effect of switch within each accent condition separately.

For the follow-up analysis of the Mandarin-accented data the effect of switch significantly improved model fit (*χ*^2^ = 48.52, *df* = 2, *p* < .001), with model estimates indicating that the within-accent switch condition had lower overall amplitude as compared with both the no switch (*ß* = −2.69, *p* = .04) and across-accent switch conditions (*ß* = −8.98, *p* < .001). The across-accent switch condition was significantly greater than the no switch condition (*ß* = 6.30, *p* < .001), as in the primary analyses. The interaction of switch with the linear polynomial term was nonsignificant (*χ*^2^ = 2.75, *df* = 2, *p* = .25), indicating similar rates of increase for all three conditions. There were some differences in shape, particularly for the across-accent switch condition, which were captured by significant interactions of switch with the quadratic and cubic polynomials (respectively: *χ*^2^ = 21.40, *df* = 2, *p* < .001; *χ*^2^ = 6.14, *df* = 2, *p* < .05). The across-accent switch condition had a slightly flatter (*ß* = −56.83, *p* < .001) and earlier peak (*ß* = 28.82, *p* < .05) than the no switch condition.

The effect of switch was also significant in the follow-up analysis of the Turkish-accented data (*χ*^2^ = 116.11, *df* = 2, *p* < .001), though the relationships between each of the three levels differed from those found in the Mandarin-accented data. As predicted, the within-accent switch (*ß* = 10.29, *p* < .001) and across-accent switch (*ß* = 13.41, *p* < .001) conditions prompted larger pupil responses than the no switch condition. The interactions of switch with each of the polynomial terms were significant (all *p*s < .05), and indicated that, as compared with the no switch condition, the within-accent switch condition had a flatter peak (*ß* = −42.80, *p* = .003) and the across-accent condition increased at a faster rate (*ß* = 12.50, *p* < .001).

### Discussion

The results of Experiment 1 indicate that switching between speakers of different L2 accents is consistently more cognitively demanding than switching between speakers of the same L2 accent. Specifically, switching between speakers of different accents (either Mandarin-to-Turkish accent or Turkish-to-Mandarin accent) was more cognitively challenging than switching between speakers of the same accent (either between the two Mandarin-accented speakers or between the two Turkish-accented speakers). This outcome aligns with a recalibration distance account and active control model, in which the perceptual distance between two speakers’ productions determines the size of associated switching costs. In other words, because there are typically shared influences from a given L1 on L2 speakers’ productions, recalibrating is less cognitively demanding on within-accent switch trials. This conclusion aligns with the results of similar research that has documented an interlanguage speech intelligibility benefit; for example, a listener with an L1 Korean language background will typically understand L2 Korean-accented English better than a listener with an L1 English background (Bent & Bradlow, 2003).

Notably, this outcome indicates that the lack of an across-accent switch cost for L2-to-L1 accent switch trials in McLaughlin et al. (2023) may have been due to the familiarity of L1 accent. On this view, recalibration to an L1-accented speaker is afforded a “familiarity benefit” because the mapping for L1 accent is already well-established and easily recalled by the listener (Magnuson et al., 2021).

The results of Experiment 1 cannot definitively rule out an effect of accent difficulty. Although we selected the speakers based on prior data with the intention of matching them for difficulty (and confirmed similar perceptual distances within-accent, see Supplemental Materials), in the present experiment the actual intelligibility of the Mandarin-accented speakers were 94% and 93% while the intelligibility of the Turkish-accented speakers were 98% and 99%. Overall, the intelligibility of a given trial was negatively related to the magnitude of the pupil response (i.e., less intelligible sentences elicited more cognitive load), mirroring prior word recognition research with L2 accent and pupillometry (Porretta & Tucker, 2019); to the best of our knowledge, this study is the first to demonstrate a similar relationship for sentence-length materials using pupillometry. Further, even when statistically accounting for this difference in intelligibility, the pupil response for Mandarin accent was significantly greater. Given this difference in difficulty between the L2 accents, it is plausible that some adjustments based on the differences in overall accent difficulty contributed to the across-accent switching effect. However, based on the accent difficulty account one would expect an *asymmetry* in across-accent switches, where switching from the harder to the easier condition is less challenging than switching from the easier to the harder condition. This type of asymmetry was not present in the data, indicating that across-accent switches posed an equal cost regardless of the direction of the change between the Mandarin and Turkish accent conditions. Thus, the results of Experiment 1 do not appear to support the accent difficulty account.

Surprisingly, results of Experiment 1 also indicated that the cognitive load from switching between speakers of the same accent only increased (as compared with not switching) for the Turkish accent condition, and not the Mandarin accent condition; for the Mandarin accent condition, trials with switching within-accent actually resulted in smaller pupil responses than trials with no switch (although the size of this effect was relatively small). This outcome was not predicted, and contradicts our assumption based on the results of McLaughlin et al. (2023) that any kind of switch between speakers—whether within or across accents—will result in a processing cost compared with hearing the same speaker from the previous trial.

## Experiment 2

One important difference between McLaughlin et al. (2023) and Experiment 1 of the current study is that the former presented a mixture of (easy) L1 and (more challenging) L2 accents, while the latter presented exclusively L2 accents. These contexts may have impacted the switching costs observed in each respective investigation. In Experiment 2, we examine whether a more challenging block-wide listening context results in an upregulation of cognitive resources. With that in mind, we address three questions: (1) Does block-wide listening context impact global upregulation of cognitive resources? (2) Do the cognitive demands of L2 accent processing differ in a block-wide context where a second L2 accent is most likely (i.e., as in Experiment 1 of the present study) versus a context where an L1 accent is most likely (i.e., as in McLaughlin et al., 2023)? (3) Does global upregulation, if observed, reduce the cognitive resources required to resolve local talker and accent changes?

In Experiment 2, participants completed a block that was primarily L1 (American English) accent and a block that was primarily L2 (Hindi) accent. In addition to the primary accent, both blocks contained a subset of trials that presented a different L2 (Mandarin) accent (matched in difficulty to the L2 Hindi accent, details in Method). There were three primary analyses, relating, respectively, to the research questions above. The first pertains to whether overall cognitive engagement differed between the mostly-American block and the mostly-Hindi block. We examine whether the demand induced by the accent blocks differed in an exploratory analysis of baseline pupil diameter (i.e., pre-stimulus pupil diameter) predicting that the block-wide context manipulation induced different levels of demand that lead to global upregulation in the mostly L2-accented block.

The second analysis pertains to pupil response for the critical Mandarin accent trials. By comparing pupil response for these trials across blocks, we can determine whether the cognitive load required to process an L2 accent depends on the block-wide listening context. We predicted that a block-wide listening context of mostly-American accent or mostly-Hindi accent would affect the cognitive demands of speech processing for the L2 Mandarin accent. Specifically, the pupil response would be smaller for the critical Mandarin accent trials presented in the mostly-Hindi accent block compared with the mostly-American accent block, signaling that listeners upregulated allocation of cognitive resources for the block containing more L2 accent.

The third analysis pertains to switching effects—specifically for the accent that is more common in each block (i.e., American accent in the mostly-American accent block; Hindi accent in the mostly-Hindi accent block). For the effect of switching, there were three conditions of interest, as determined by the speakers on the current and previous trials: No switch (e.g., Hindi Talker 1 to Hindi Talker 1; American Talker 1 to American Talker 1), within-accent switch (e.g., Hindi Talker 1 to Hindi Talker 2; American Talker 1 to American Talker 2), and across-accent switch (e.g., Mandarin Talker 1 to American Talker 1; Mandarin Talker 1 to Hindi Talker 1). Here, we predicted that block-wide context would reduce switching costs in the mostly-Hindi accent block (matching Experiment 1) as compared with the mostly-American accent block (matching McLaughlin et al., 2023). Relatedly, increasing switch frequency has been shown to reduce the cost of switching between tasks (e.g., Dreisbach & Haider, 2006; Kang & Chu, 2021; Siqi-Liu & Egner, 2020). These predicted outcomes would indicate that global upregulation (induced by block context) subsequently reduced the cognitive resources required to resolve local talker and accent changes.

### Method

Preregistration of Experiment 2 is available online (https://osf.io/ypdwg).

#### Participants

Sixty-two young adult participants (46 women, 16 men; Age *M* = 19.03, *SD* = 1.14) were recruited from Washington University’s psychology participants pool and participated for course credit. After replacing subjects who were excluded due to experiment or equipment malfunction (five participants) and blinking-related data loss (three participants), and for being under our minimum age requirement (three participants), 51 participants remained (37 women, 14 men; Age *M* = 19.12, *SD* = 1.11). All subjects were L1 speakers of English (of an American dialect) with normal hearing, normal (or corrected-to-normal) vision, and reported minimal exposure to Mandarin Chinese and Hindi (e.g., did not live with a speaker of either of those languages or study either of those languages in school). All participants reported learning at least one additional language in school; of the 51 total participants, approximately 82% reported learning their additional language before or at the age of 12 and approximately 24% reported learning their additional language before or at the age of 5 (at home).

#### Materials

**Fig. 4:**
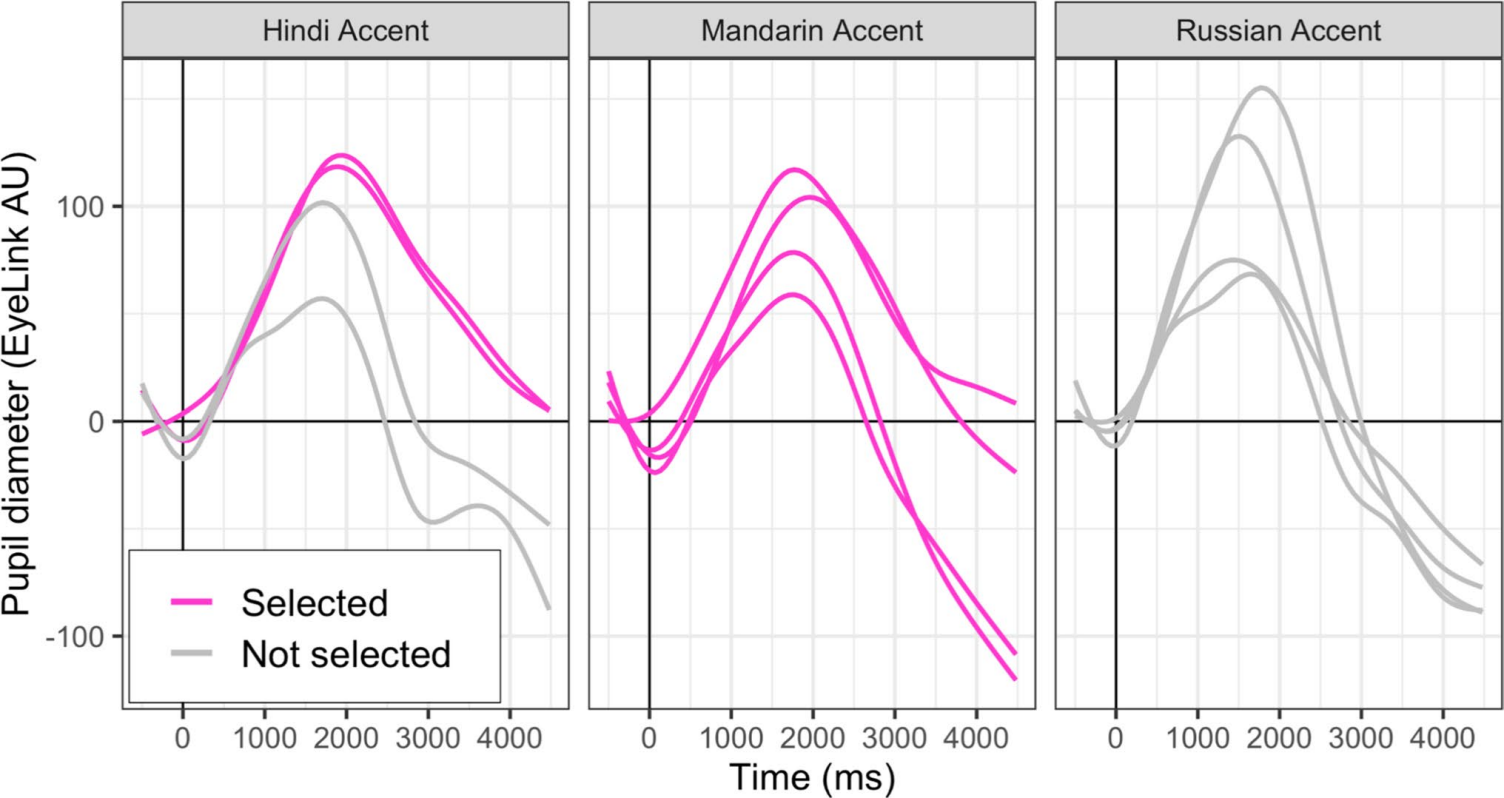
Pilot data are shown with predicted fit lines from the “geom_smooth()” function in *ggplot2*. Solid vertical line at zero indicates sentence onset and solid horizontal line at zero indicates baseline. Stimuli from two Hindi-accented speakers of English (pink) were selected for use in the present experiment along with the four Mandarin-accented speakers of English (pink). An additional two Hindi-accented speakers (gray) and four Russian-accented speakers (gray) were piloted in the same task. The Russian L2 accent was considered as an alternative for the Mandarin accent, but ultimately not used in Experiment 2 because the range in pupil response to the four speakers was greater. (Color figure online)

The materials from Experiment 2 were identical to Experiment 1, except for the following differences. As in Experiment 1, the stimuli used in Experiment 2 were recordings of HINT sentences (Nilsson et al., 1994) from SpeechBox (Bradlow, n.d.). The speaker set comprised two American English accented L1 speakers, two Hindi-accented L2 speakers^2^, and four Mandarin-accented L2 speakers of American English.^3^ All talkers were men. Talkers were selected based on a pilot study that was conducted to confirm that pupil responses (indexing cognitive load) for the L2 accented speakers were similar at baseline (details on method of pilot study are available in the Supplemental Materials). Figure 4 presents an overview of the pilot study’s data, highlighting the L2-accented speakers selected for use in Experiment 2. Details on the pilot can be found in the Supplemental Materials.

The experiment was split into two 60-trial blocks. One block was mostly (i.e., 44 trials) American-accented trials, and the other was mostly (i.e., 44 trials) Hindi-accented trials. In each block, there were also 16 Mandarin-accented trials. For each of these accents, there were two distinct speakers per block. Note that the two Mandarin speakers did not repeat across blocks. For example, if Mandarin Talkers 1 and 2 were in the mostly-American accent block, then Mandarin Talkers 3 and 4 would be in the mostly-Hindi accent block. We counterbalanced both which set of Mandarin talkers were presented in each block and which of the two blocks was presented first.

Speakers were presented in a pseudo-random order within each block to maintain a fixed number of each type of switch. The first trial in each block was an American accented speaker in the mostly-American accent block or a Hindi accented speaker in the mostly-Hindi accent block. The first trial was not included in the analyses of switch effects as it was neither a switch trial nor a repeat trial. Thus, there were 43 trials per block of the more common (block-wide context) accent that were analyzed when examining switch effects. Among these 43 trials, 14 were no switch trials (seven per speaker), 14 were within-accent switch trials (seven per speaker), and 15 were across-accent switch trials (either seven or eight per speaker). In each block, there were 16 Mandarin talker trials (eight per speaker), all of which were across-accent switches (i.e., either American English to Mandarin in the mostly-American accent block or Hindi to Mandarin in the mostly-Hindi accent block). Half of the Mandarin speaker trials were set to appear in the first 30 trials of a given block while the other half were set to appear in the last 30 trials of the block.

#### Procedure

The procedure of Experiment 2 was identical to Experiment 1, except for the following differences: (1) Rather than a four-trial practice phase, one practice trial was integrated into the instructions, and (2) Experiment 2 used two blocks rather than four blocks, resulting in only one self-timed break.

##### **Data preparation**

All data preparation procedures matched those detailed in Experiment 1. For the assessment of intelligibility, the eight talkers were all between 97% and 100% intelligible. The average scores per speaker were: 100% for American-English-accented Speaker 1, 99.7% for American-English-accented Speaker 2, 98.1% for Hindi-accented Speaker 1, 97.7% for Hindi-accented Speaker 2, 99.2% for Mandarin-accented Speaker 1, 98.6% for Mandarin-accented Speakers 2 and 3, and 98.1% for Mandarin-accented Speaker 4.

### Results

#### Effects of block-wide listening context on baseline pupil diameter

**Fig. 5:**
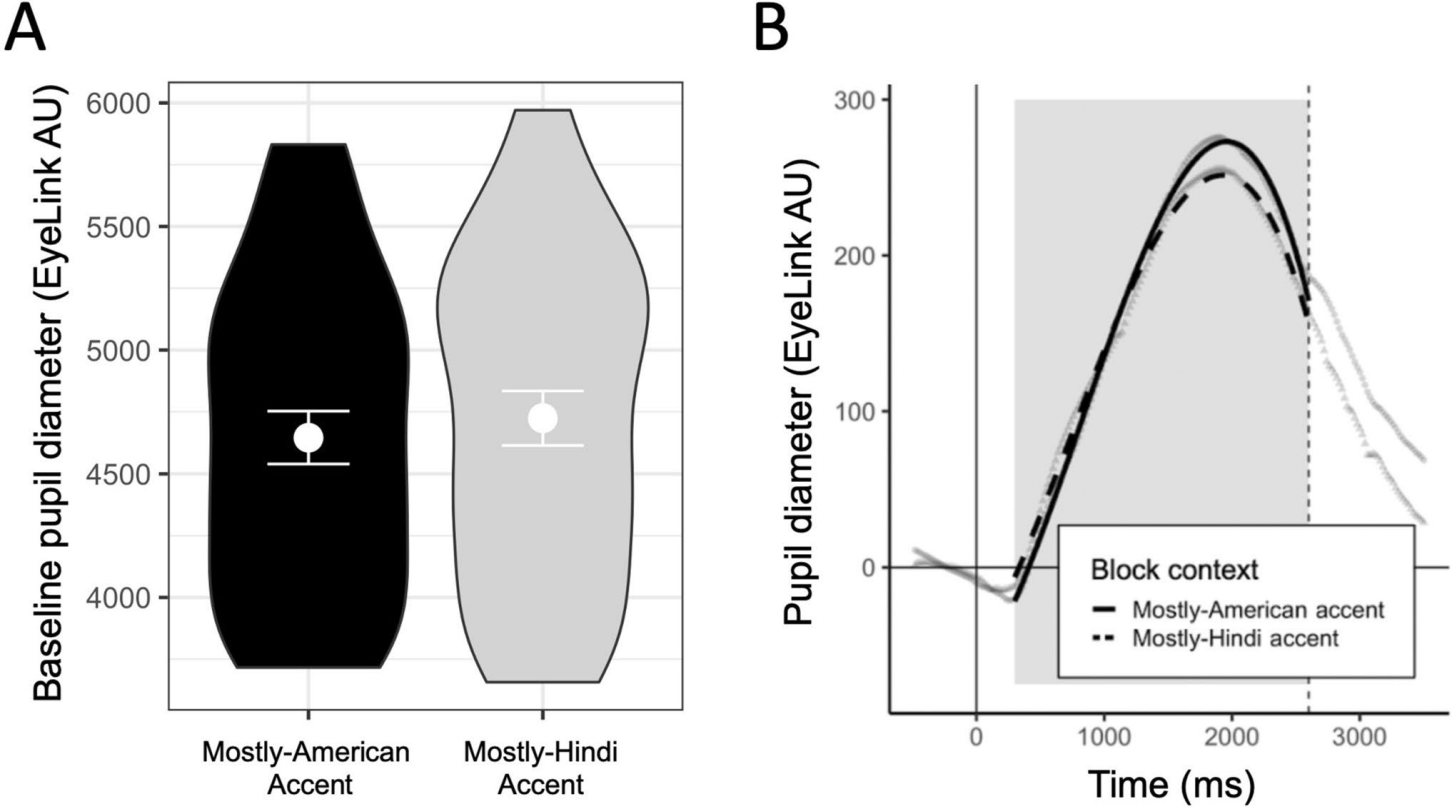
The effects of block-wide context on baseline pupil diameter and perception of Mandarin accent. **A** Violin plots show density distributions of participant means with group mean points and standard error bars overlaid. **B** Points represent mean values and lines represent predicted model fits. The solid vertical line at zero indicates sentence onset and dashed vertical line indicates average sentence offset. The gray boxes denote the areas of data used for analyses

We began by exploring the effects of block-wide listening context on baseline pupil diameter. If participants are using the block-wide listening context to predict and prepare for upcoming trials, that difference between blocks should be reflected in pupil diameter before stimulus onset. This set of analyses was not preregistered. Pupil data from the baseline period (−500 ms to 0 ms) used for aligning the task-evoked data for analysis was averaged by-trial within each subject. A linear mixed-effects model was fit with random intercepts by subject and item and random slopes of block context by subject. Block context was the only fixed effect examined in the model. Estimates indicated a marginal difference in baseline pupil diameter by block context (*ß* = 78.40, *p* = .07). As shown in Fig. 5A, baseline pupil diameter was slightly, but nonsignificantly, greater in the mostly-Hindi accent block compared with the mostly-American accent block.

#### Effect of block-wide listening context on global perception of L2 (Mandarin) accent

Next, we examined the effects of block-wide listening context on cognitive load for perception of Mandarin accent. All log-likelihood model comparisons from the block-wide context analysis of Experiment 2 are reported in Table 2. Growth curve analysis was used to fit the shape of the task-evoked pupil response with linear, quadratic, and cubic polynomials (all significantly improved fit; *p*s < .001). The effects of intelligibility and trial both accounted for a significant amount of variance (*p* = .01 and *p* < .001, respectively) and were thus included in the model. As in Experiment 1, poorer intelligibility predicted larger amplitudes (*ß* = −27.62, *p* = .01) and pupil response amplitude decreased across the task (*ß* = −1.92, *p* < .001).

**Table 2:** Log-likelihood model comparisons for block-wide context analysis of Experiment 2

Effect	*χ*^2^	df	*p*
Linear polynomial	25.89	1	<.001
Quadratic polynomial	38.47	1	<.001
Cubic polynomial	15.31	1	<.001
Intelligibility	6.09	1	.01
Trial	6319.40	1	<.001
Block context	24.98	1	<.001
Block context : Linear polynomial	49.47	1	<.001
Block context : Quadratic polynomial	4.13	1	.04
Block context : Cubic polynomial	2.93	1	.09

Colons indicate interactions. Levels of block context included mostly-American accent (reference) and mostly-Hindi accent.

The fixed effect of block context (levels: mostly-American accent, mostly-Hindi accent) and its interaction with each polynomial term was examined for the Mandarin accent trials. Block context (*χ*^2^ = 24.98, *df* = 1, *p* < .001) and its interaction with the linear and quadratic polynomials (*p*s < .05) significantly improved model fit. As shown in Fig. 5B, the pupil response for Mandarin accent was greater in the mostly-American block than the mostly-Hindi block, with a higher and sharper peak pupil response.

#### Effect of block-wide listening context on resolving local talker and accent changes

Lastly we examined the effects of block-wide listening context on cognitive load for switching within- and across-accents. In the switching analysis, log-likelihood model comparisons (Table 3) indicated that the linear, quadratic, and cubic polynomials all improved model fit (*p*s < .01). Effects of trial and intelligibility also improved model fit (*p*s < .001) and were thus included in the model. The effect of accent (levels: American, Hindi) on the intercept was nonsignificant (*χ*^2^ = 2.56, *df* = 1, *p* = .11), but the effect of accent on the linear (*χ*^2^ = 26.95, *df* = 1, *p* < .001) and cubic (*χ*^2^ = 11.00, *df* = 1, *p* < .001) terms improved model fit (effect on the quadratic polynomial: *p* > .05). Model estimates indicated a faster rate of increase of the pupil response (*ß* = 40.61, *p* < .001) and a delayed peak of the pupil response (*ß* = −82.58, *p* < .001) for the Hindi accent, as compared with the American accent.

**Table 3:** Log-likelihood model comparisons for analysis of switching effects in Experiment 2

Effect	*χ*^2^	*df*	*p*
Linear polynomial	8.34	1	.004
Quadratic polynomial	35.73	1	<.001
Cubic polynomial	10.48	1	.001
Intelligibility	121.66	1	<.001
Trial	4741.40	1	<.001
Accent	2.56	1	.11
Switch	48.52	2	<.001
Accent : Switch	38.69	2	<.001
Accent : Linear polynomial	26.95	1	<.001
Accent : Quadratic polynomial	2.70	1	.10
Accent : Cubic polynomial	11.00	1	<.001
Switch : Linear polynomial	3.45	2	.18
Switch : Quadratic polynomial	0.25	2	.88
Switch : Cubic polynomial	7.08	2	.03
Accent : Switch : Linear polynomial	0.69	2	.71
Accent : Switch : Quadratic polynomial	3.70	2	.16
Accent : Switch : Cubic polynomial	0.38	2	.83

Colons indicate interactions. Levels of accent included Mandarin (reference) and Hindi. Levels of switch included no switch (reference), within-accent switch, across-accent switch.

**Fig. 6:**
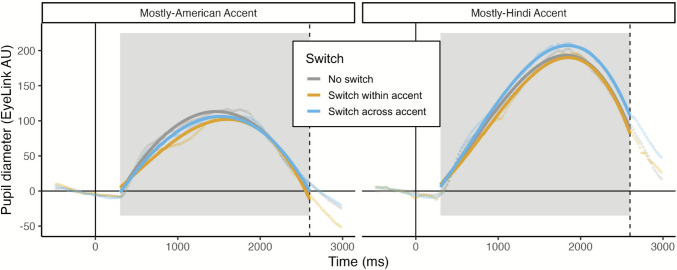
The effects of block-wide context on switching are shown with raw data points and predicted fit lines. The solid vertical line at zero indicates sentence onset and dashed vertical line indicates average sentence offset. The gray boxes denote the areas of data used for analyses. (Color figure online)

The effect of switch (*χ*^2^ = 48.52, *df* = 1, *p* < .001) and the interaction of switch with accent (*χ*^2^ = 38.69, *df* = 1, *p* < .001) both improved model fit. As shown in Fig. 6 across-accent switch trials resulted in larger pupil responses than no switch trials in the mostly-Hindi accent block (*ß* = 12.92, *p* < .001), but not the mostly-American -block (*ß* = 2.47, *p* = .17). In other words, switching across accents from Mandarin to Hindi was cognitively costly while switching across accents from Mandarin to American was not. The asymmetry between across accent switches into L1 accents and into L2 accents is consistent with the finding from McLaughlin et al. (2023). Switching between speakers of the same accent also elicited a larger pupil response overall than not switching (*ß* = 6.18, *p* < .001), although this effect did not differ significantly based on block-wide context (*ß* = −1.54, *p* = .56). Lastly, there was an interaction of the effect of switch and the cubic polynomial term (*χ*^2^ = 7.08, *df* = 1, *p* = .03); model estimates suggested that trials with a switch within accent slowed the pupil response to a greater degree than no switch trials (*ß* = −31.20, *p* = .03). Three-way interactions of accent, switch, and each polynomial term did not improve model fit (*p*s > .05).

As in Experiment 1, to best understand the nature of the accent by switch interaction we examined the effect of switch within each accent condition separately. Effects of intelligibility and trial number were included in the follow-up models, as in the primary models. Within the Hindi accent condition, both within-accent and across-accent switch trials resulted in larger overall pupil response amplitude than no switch trials (*ß* = 21.67 and *ß* = 23.93, respectively; *p*s < .001). Rotation of the levels of switch revealed that the within-accent and across-accent conditions did not differ (*ß* = 2.26, *p* = .21). The levels of switch also did not interact with any of the polynomial terms (*p*s > .05), indicating a similar shape of the pupil response function across conditions. In contrast, within the American accent condition neither within-accent nor across-accent switch trials resulted in a difference in overall pupil response amplitude than no switch trials (*p*s > .05); interactions with the polynomial terms were also nonsignificant (*p*s > .05). Thus, although the primary analyses did not indicate a significant interaction for the difference of no switch and within-accent switch trials based on block-wide context (*p* = .56), follow-up analyses of each accent condition indicate a within-accent switch cost only in the mostly-Hindi accent block and not the mostly-American accent block.

### Discussion

In Experiment 2, participants completed a block that was primarily L1 (American English) accent and a block that was primarily L2 (Hindi) accent. In addition to the primary accent, both blocks contained a subset of critical trials that presented a different L2 (Mandarin) accent of similar difficulty. The critical finding of Experiment 2 was that the Mandarin-accented trials elicited a smaller pupil response in the mostly-Hindi accent block than in the mostly-American accent block. Exploratory analyses of baseline pupil diameter also indicated slightly (though nonsignificantly) larger pupil size in the mostly-Hindi accent block, which may provide insight into the mechanism by which listeners made these adjustments in a mostly-L2 listening context: Listeners may have upregulated cognitive resources depending on the difficulty of the block-wide listening context. We replicated the general finding from McLaughlin et al. (2023), such that across-accent switches into an L2 accent were particularly costly while across-accent switches into an L1 accent were not as costly. Surprisingly, however, the costs associated with accommodating talker and accent changes (i.e., trial-to-trial switching costs) were not uniformly reduced by globally upregulating resources across the mostly-Hindi accent block.

To the best of our knowledge, this study is the first to demonstrate that a predominantly L2 accent listening context can prompt upregulation of cognitive resources and subsequently reduce global processing costs for L2 accent perception. Although prior studies have examined benefits of multi-accent training for understanding novel (i.e., untrained) L2 accent (e.g., Baese-Berk et al., 2013), focus has primarily been on intelligibility scores (i.e., transcription accuracy) during posttest sessions. In contrast, Experiment 2 presents evidence of the benefits of an L2 (Hindi-accented English) listening context for reducing the online perceptual processing costs of another L2 accent (Mandarin Chinese–accented English), where both accents were presented in a single, intermixed session.

However, it should also be noted that all Mandarin-accented trials were across-accent switches. We chose to design our blocks with that constraint to make sure there were enough no switch, within-accent switch, and across-accent switch trials within each block. While the observed difference for Mandarin trials (i.e., reduced pupil response in the mostly-Hindi accent block compared with the mostly-American accent block) indicates that the block-wide listening context affected speech perception, we cannot necessarily isolate block-wide upregulation as the sole cause of the difference in cognitive load on Mandarin-accented trials between blocks. Because the *N* − 1 trial was always an across-accent switch, the observed effect was plausibly partially driven by trial-to-trial adjustments. As found in McLaughlin et al. (2023), there was an asymmetry in the cost of shifting from an L1 accent to an L2 accent compared with shifting from an L2 accent to an L1 accent. Because the Mandarin trials were exclusively across-accent switches, it cannot be ruled out that the increased cost for mostly-American accent blocks was partially or entirely driven by the need to shift from an L1 accent on trial *N* − 1 to an L2 accent on trial *N* (i.e., a local, trial-to-trial adjustment). An important future direction would be to balance the number of critical transitions such that local and global mechanisms could be disambiguated.

We also examined the effect of block-wide listening context on the magnitude of switching effects, predicting that global upregulation during the mostly-Hindi accent block would subsequently reduce the cognitive resources required to resolve local talker and accent changes. The rationale behind this prediction came from the finding in Experiment 1 of the current study, whereby the predicted switching effects did not emerge in an experimental context where the trials were exclusively (more challenging) L2-accented instead of the mixture of L1- and L2-accented trials seen in McLaughlin et al. (2023). Results, however, did not indicate a reduction of switching costs in the mostly-Hindi accent block. Rather, no switching costs were observed for the mostly-American accent block. In the mostly-Hindi accent block, only across-accent switching costs—and not within-accent switching costs—were observed. Although there may have been differences in upregulation by block-wide listening context, we propose that these global differences did not necessarily impact local switching costs. Rather, it appears that switching costs may have been reduced in a block-wide listening context that was substantially less challenging. In future work, parametrically varying the difficulty of a multi-accent context (e.g., with background noise) could help to unravel the relationship between listening context, overall cognitive challenge, and associated costs for resolving talker and accent changes.

## General discussion

The aims of the two experiments of the current study were to (1) determine whether the across-accent switching costs observed in McLaughlin et al. (2023) were driven by engagement of a recalibration mechanism or differences in (L1 and L2) accent difficulty and (2) investigate the potential impact of global listening context on within- and across-accent switching costs. To address the first aim, we investigated multi-talker processing costs in a setting with multiple L2 accents that were of similar overall difficulty. Although the L2 talkers in McLaughlin et al. were highly intelligible, the more challenging L2 accent condition elicited a larger overall pupil response than the L1 accent condition (see also Brown et al., 2020; McLaughlin & Van Engen, 2020). In Experiment 1, however, we were able to isolate the potential role of an active control (i.e., recalibration) mechanism by presenting a set of two L2 Turkish-accented speakers and two L2 Mandarin Chinese-accented speakers of English of similar difficulties. We posited two potential outcomes and corresponding accounts of the data: Either within-accent and across-accent switches would not differ (*accent difficulty account*), or across-accent switches would elicit larger pupil response than within-accent switches (*recalibration distance account*).

Results of Experiment 1 aligned with the recalibration distance account, such that switching between speakers of the same accent (either between the two Mandarin-accented speakers or between the two Turkish-accented speakers) was less cognitively challenging than switching between speakers of different accents (either Mandarin-to-Turkish accent or Turkish-to-Mandarin accent). More broadly, this outcome is best accounted for by the active control model (Heald et al., 2016; Magnuson & Nusbaum, 2007; Nusbaum & Magnuson, 1997), a theoretical account that proposes that talker-switching costs stem from storing the incoming speech signal in working memory (Wong et al., 2004) and calculating a phonological mapping (i.e., recalibrating) from the current talker’s idiosyncratic productions to a listener’s phonological space (see schematic by Magnuson, 2018). The present study provides evidence that the amount of cognitive load imposed by switching between talkers corresponds with the perceptual distance between the two speakers’ productions. In other words, because the acoustic space of productions from speakers with the same L2 accent is more similar, recalibrating (or “computing a mapping”; Magnuson et al., 2021) is less cognitively demanding on within-accent switch trials.

Notable for future work, despite having matched the selected speakers chosen for the experiment based on prior-collected intelligibility scores, their intelligibility in our experiment ultimately differed. Thus, although we aimed to match the speakers for difficulty (and confirmed similar perceptual distances within-accent, see Supplemental Materials), our descriptive statistics of intelligibility within-experiment indicated that the Mandarin-accented speakers were ultimately slightly less intelligible than the Turkish-accented speakers. Further, even after statistically accounting for intelligibility scores, the Mandarin accent elicited a larger pupil response (indicative of greater cognitive load) than the Turkish accent, overall. It is possible that the unique segmental and/or suprasegmental qualities of each L2 accent contribute to the cognitive load imposed on speech processing.

In a previous study using similar methodology to the current work (McLaughlin et al., 2023), we found that switching from L1 accent to L2 accent also elicited an across-accent switching cost. The reverse, switching from L2 accent to L1 accent, however, did not impose greater costs than within-accent switching. Given the findings of the current study, we hypothesize that the lack of an across-accent switching cost for L2-to-L1 accent switch trials in McLaughlin et al. (2023) may have been due to the greater familiarity of the L1 (American) accent, which matched the accent of the (American) listeners who participated in the study. In future work, it will be critical to investigate whether familiarity with an accent may reduce the costs associated with computing a mapping. A “familiarity benefit” has been hypothesized in previous work by Magnuson et al. (2021). In that study, the authors compared the processing costs associated with talker switching for family members versus novel talkers, predicting that the characteristics of speech produced by family members ought to be well-known and easily recalled by the listener. Results, however, indicated similar talker switching costs for familiar and unfamiliar talkers. Whether familiarity with an accent may provide a benefit for across-accent switching, however, remains to be examined.

One surprising result from Experiment 1 was that greater cognitive demands for within-accent switching only emerged in the Turkish accent condition, and not the Mandarin accent condition. For the Mandarin accent condition, within-accent switch trials actually resulted in smaller pupil responses than no switch trials (although the size of this effect was relatively small). This outcome contradicts an important assumption of the current study and McLaughlin et al. (2023); namely, that any kind of switch between talkers—whether within or across accents—will result in a processing cost compared with hearing the same talker from the previous trial. We hypothesized that this result may have stemmed from an effect of the global listening context and developed Experiment 2 to test this possibility.

Experiment 2 manipulated the block-wide listening context by presenting one block that was primarily L1 (American English) accent and one block that was primarily L2 (Hindi) accent. We aimed to assess whether the global context (induced by the less challenging or more challenging accent) affected both perception of a different L2 accent (i.e., Mandarin) and local switching costs for the primary accent in each block. Indicating potential evidence for greater global upregulation of cognitive resources in the mostly L2 block, we found that pupil diameter was marginally larger at baseline (i.e., before stimulus onset). Given some evidence for greater upregulation in the mostly Hindi accent block, we then assessed whether that upregulation affected both (a) the overall demands of processing Mandarin-accented speech and (b) the local costs of switching between talkers of the same and different accents. Critically, the amplitude of the pupil response for Mandarin trials differed by block, indicating that being in a block-wide listening context with mostly L2 speech reduced the difficulty resolving L2 speech from a different accent. Against our prediction, the global upregulation in the mostly-Hindi accent block did not reduce the size of local switching costs. We note, however, that a stronger manipulation—inducing greater global upregulation—could possibly reduce local switching costs. Alternatively, it may be the case that these local switching costs are fully driven by trial-to-trial factors, and the global upregulation induced by the block-wide listening context will not affect them. These are important areas for future research.

### Future directions

Throughout the present experiments and the experiments in McLaughlin and colleagues (2023), we explored whether trial-to-trial adjustments across accents are driven by tuning to accent-specific features or to the relative difficulties of the accents themselves. While these studies have examined L1 versus L2 accents for across-accent talker switches, future work could expand on our findings by examining rapid adjustments for varieties of L1 dialect, which we predict would demonstrate similar effects but on a smaller scale (assuming that perceptual distances are smaller between the given L1 dialects than between an L1 and L2 accent). Were this the case, it would further strengthen the recalibration distance account. Additionally, further probing of the effect of global difficulty/context could take an alternative approach by parametrically manipulating the challenge of speech perception independent of the accent manipulation. For example, future work should explore how recent experience with adverse listening conditions may prompt upregulation of cognitive resources and subsequently reduce the demands of speech processing. One possibility would be to degrade the spectral quality of a speech signal via noise-vocoding or background noise (Mattys et al., 2013). If global upregulation across the block helps a listener prepare for difficulty, one would predict that difficulty imposed by one manipulation (e.g., increased noise) could reduce local cognitive load for another type of listening challenge (e.g., talker accent). This prediction differs, but is not mutually exclusive, from the recalibration distance account, which would predict that sequence effects should not generalize across factors that result in similar distance in acoustic space between speakers.

An interesting parallel can be drawn between our design and the “asymmetrical switch cost” finding in the traditional task switching literature (Allport et al., 1994). When switching between tasks of different difficulty, it has been theorized that the harder task interferes with the easier task, as greater inhibition is needed to switch away from the harder task. This difficulty inhibiting the difficult task set can lead to a larger switch cost (i.e., difference in reaction time or error rate between task repetitions and task switches) when switching from the difficult to easy task compared with switching from the easy to difficult task. Interestingly, we did not see this same type of pattern for switching from the more difficult accent to the easier accent in the current study (or in the experiments of McLaughlin et al., 2023). One possible reason that this pattern did not emerge is that the task (i.e., listen to a sentence and repeat it aloud) remained consistent (though see Schneider & Anderson, 2010; Spitzer et al., 2019, for accounts of asymmetry being driven by sequential difficulty or task strength rather than inhibiting a task set). Alternatively, there may be important differences between the control processes used to inhibit a task set and the active control needed to “recalibrate” for accent/talker changes. The current study and McLaughlin et al. (2023) provide a first step in investigating the role of previous sequential experience on current trial performance, but this and many other open questions remain for future studies.

Another critical question that remains to be addressed is whether there is a threshold at which recalibration becomes suboptimal, and—if so—what alternative mechanism(s) may be recruited to support speech processing under such circumstances. The most extreme case, for example, would be a block in which every single trial presents a novel talker or accent. In this scenario, would the listener continue to engage a recalibration mechanism to accommodate talker or accent changes on every trial? A more efficient alternative mechanism that would not be mutually exclusive is *criteria relaxation* (see discussion in Zheng & Samuel, 2020). On this view, listeners may relax their thresholds for accepting input as a particular phoneme (or lexical item, etc.), thus allowing a wider range of acoustic realizations of an item to map onto a given category label. Supporting this idea, Babel and colleagues (2021) leveraged a natural difference in familiar (devoiced /z/) vs. unfamiliar (voiced /s/) accent variation in North American English, determined via corpus data. L1 English listeners were able to accommodate both voicing and devoicing (as determined by endorsement of items in a lexical decision task), but /z/-devoicing resulted in a directional category shift while /s/-voicing resulted in a relaxation of the category. Thus, these results indicated that different adaptive mechanisms may be recruited under different circumstances to accommodate spoken language variation. It remains to be determined whether a criteria relaxation mechanism can be employed in a multi-talker and multi-accent setting, and at what point it would be more optimal than a recalibration mechanism.

### Conclusion

Alternating between different talkers during listening typically incurs a cognitive processing cost. The present study examined how these processing costs manifest, and how they may be affected by global listening context. Across two experiments, we investigated (1) whether talker and accent switching costs are driven by engagement of a recalibration mechanism, and (2) whether global listening context affects the overall demands of processing L2 accent and/or the magnitude of talker and accent switching costs. The results of our first experiment supported the conclusion that accommodation of talker and accent switching is supported by a recalibration mechanism, such that the perceptual distance between two speakers’ productions determines the size of associated switching costs. In our second experiment, we examined whether a more challenging block-wide listening context (i.e., a block containing all L2-accented speakers) would cause a global upregulation of cognitive resources, thus reducing the cognitive resources required to (a) process L2 accent, and (b) resolve local talker and accent changes. Exploratory analyses suggested that global upregulation may have reduced the overall cognitive demands of processing L2 accent but did not affect the cognitive demands of resolving talker and accent switching. Returning to the dinner party example from the introduction, we conclude that global upregulation during multi-talker listening may reduce the challenge of accent perception, but that upregulation may not be able to reduce the costs associated with managing switches between talkers and accents.

## Supplementary Information

Below is the link to the electronic supplementary material.

Supplementary file1 (DOCX 272 KB)

## Footnotes

1 The speaker identification numbers were ALL_043 (Mandarin-accented speaker 1), ALL_032 (Mandarin-accented speaker 2), ALL_091 (Turkish-accented speaker 1), and ALL_092 (Turkish-accented speaker 2).

2 Although the database that the Hindi materials were sourced from Bradlow (n.d.) label the Hindi speaker as having an “L2 accent”—meaning that English would have been learned as a secondary language following Hindi—we acknowledge that there is potential ambiguity as to whether the Hindi-accented speaker has L2 accent or what we would more simply classify as “foreign accent.” The latter would refer to a speaker who learned English as an L1, but speaks with a regional dialect that is stereotyped by American listeners as foreign, nonetheless. We do not have demographic or detailed language background information about the Hindi speaker to definitively rule out the latter classification, but for the sake of simplicity label the speaker as L2-accented in the present study. Given the results of the Supplemental machine learning analysis of the stimuli, we can conclude that the talker meets the needs of the experiment (a “foreign” accent that is perceptually distant from the other foreign accent, Mandarin Chinese-accented English).

3 The speaker identification numbers were ALL_066 (American English 1), ALL_133 (American English 2), ALL_034 (Hindi 1), ALL_118 (Hindi 2), ALL_016 (Mandarin 1), ALL_021 (Mandarin 2), ALL_032 (Mandarin 3), and ALL_037 (Mandarin 4).

## Declarations

### Open Practices Statement

The data and materials for both experiments are available from online (https://osf.io/gd8rp/files/osfstorage). Preregistration of Experiment 1 is available online (https://osf.io/8y4hg). Preregistration of Experiment 2 is available from: https://osf.io/ypdwg
