# Supplementary material for: Sequence effects during speech perception reveal multi-accent processing costs
Source: Atten Percept Psychophys. 2026 Feb 23;88(3):83. doi: 10.3758/s13414-025-03220-5 (PMC12929306; doi:10.3758/s13414-025-03220-5)
Supplement: Supplementary file 1 — Supplementary file1 (DOCX 272 KB) [file 13414_2025_3220_MOESM1_ESM.docx]

**Supplemental Materials**

**Section A**

In Supplemental Materials Section A, we report information about the method of the pilot study used to select talkers for Experiment 2. Four Hindi-, four Mandarin-, and four Russian-accented male speakers were included in the pilot. We aimed to select two talkers with either Hindi- or Russian-accented productions who were fairly well-matched (as determined by pupil response/cognitive load) to the four selected Mandarin talkers.

**Participants**

Twenty-four young adult participants (15 female, 9 male; Age M = 19.79, SD = 1.02)

were recruited from the Washington University Psychology Participants Pool. No participants were excluded. All subjects were L1 speakers of English with normal hearing, normal (or corrected-to-normal) vision, and had minimal exposure to Mandarin Chinese, Hindi, and Russian.

**Materials**

As in Experiments 1 and 2, stimuli for the pilot study were sourced from SpeechBox (Bradlow, n.d.). Four talkers per accent (Hindi, Mandarin, and Russian) were selected from SpeechBox based on the researchers’ subjective impressions of accent strength and intelligibility. The speaker identification numbers for each accent were as follows: Hindi-accented talkers ALL_034, ALL_117, ALL_118, and ALL_149; Mandarin-accented talkers ALL_016, ALL_021, ALL_032, and ALL_037; and Russian-accented talkers ALL_010, ALL_140, ALL_142, and ALL_144. Target sentences for the stimuli come from the Hearing in Noise Test (HINT; Nilsson et al., 1994), and were all semantically and syntactically normal. Ten unique sentences were selected per talker in the task, resulting in a total of 120 unique sentences.

**Procedure**

The general pupillometry procedure matched Experiments 1 and 2. However, for the accent pilot items were randomly (not pseudo-randomly) intermixed across participants. All participants were presented with all 120 items split into two 60-trial blocks with a self-timed break in between. Participants were instructed to remain in the headrest during the break.

Data were prepared using the same steps as reported for Experiments 1 and 2: blink identification, expansion, and interpolation; smoothing; and baselining.

**Section B**

In Supplemental Materials Section B, we report information about how perceptual distance was calculated post-hoc for talkers in Experiments 1 and 2. A self-supervised machine learning tool (Chernyak et al., 2024; Hsu et al., 2021) was used to compare pairs of sentences produced by each talker. The Perceptual Similarity Space for Speech-Pairwise Distance Matrix (PS3-PDM) tool takes two audio files at a time and calculates pairwise distances of the corresponding trajectories in Hidden-Unit Bidirectional Encoder Representations from Transformers (HuBERT; Hsu et al., 2021) embedding space. Assuming that the same linguistic context is present in each file (e.g., the sentence “the gray mouse ate the cheese”), pairs of talkers can be compared to determine the holistic distance in acoustics; this distance measure is independent of defined acoustic-phonetic features, and has been shown to predict L1 and L2 talker intelligibility more thoroughly than traditionally-defined linguistic cues.

For Experiment 1, the aim of calculating HuBERT distances was to confirm that the perceptual distance between two talkers of different L1 backgrounds (such as Mandarin-accented vs. Turkish-accented) was greater than those of the same L1 background (such as two Mandarin-accented talkers and two Turkish-accented talkers, respectively). A total of 100 sentences produced in isolation by each talker were compared, resulting in 100 distance measures for the Mandarin-Mandarin comparison (orange box plot of Figure 1S), 100 for the Turkish-Turkish comparison (white box plot of Figure 1S), and 400 for the Mandarin-Turkish comparison (gray box plot of Figure 1S). Significant differences between comparison types were determined with a linear model, using the lme4 and lmerTest packages in R.

For Experiment 2, our focus was on the perceptual distances between pairs of talkers within a given block type. A total of 59 sentences produced in isolation were available for all talkers in the experiment. For the mostly American accent block, we compared the two American-accented talkers to each other (59 observations), and then each American-accented talker to each Mandarin-accented talker (472 observations); for the mostly Hindi accent block, we compared the two Hindi-accented talkers to each other (59 observations), and then each Hindi-accented talker to each Mandarin-accented talker (472 observations). The critical comparison, shown in Figure 2S, was “within-accent” (red box plots) versus “across-accent” (blue box plots) distances. In both blocks, the across-accent HuBERT distances were significantly greater. Significant differences between comparison types were determined with separate linear models for each block type, using the lme4 and lmerTest packages in R.

**Figure 1S**


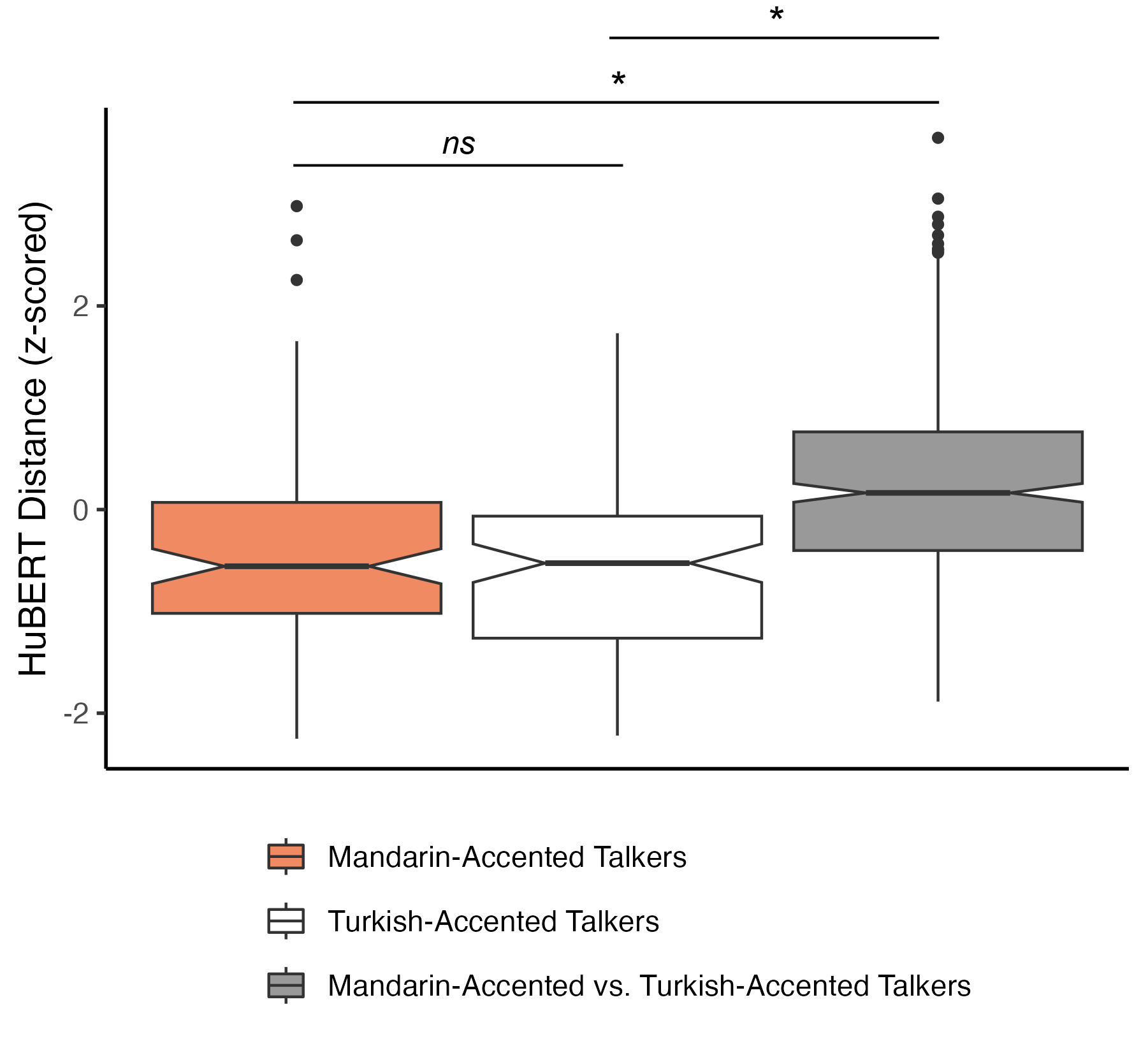


*Note.* Perceptual distances between pairs of talkers in Experiment 1 are shown with box plots. HuBERT distances were calculated for each of 120 sentences for each talker pair. The distances across-accent (between the Mandarin-accented and Turkish-accented talkers; gray box) were significantly greater than the distances “within-accent” (Mandarin-accented talker A vs. B; Turkish-accented talker A vs. B).

**Figure 2S**


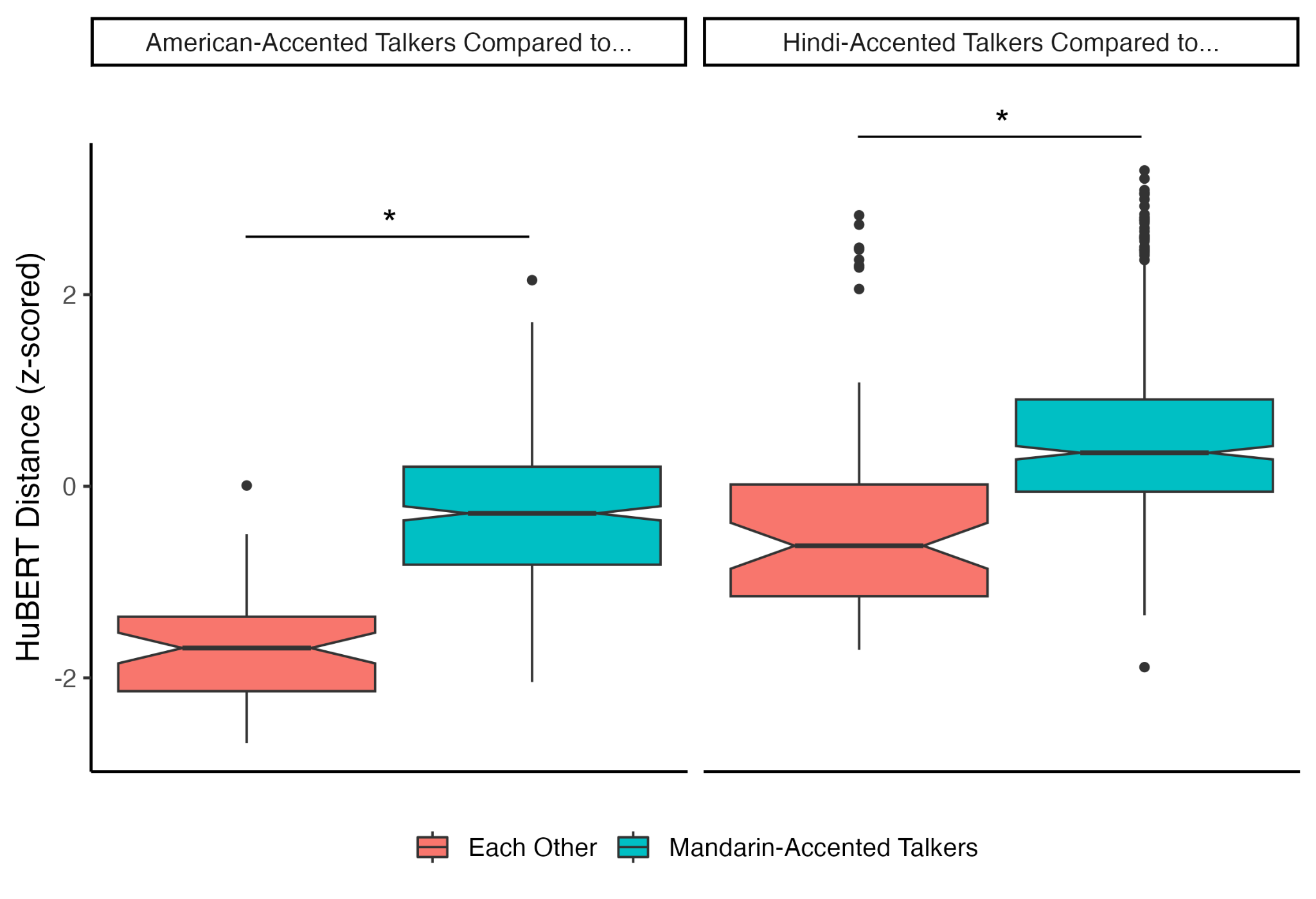


*Note.* Perceptual distances between pairs of talkers in Experiment 2 are shown with box plots. HuBERT distances were calculated for each of 59 sentences for each talker pair. The distances across-accent were significantly greater than the distances “within-accent” in each block type.
